# Bioturbation of peanut worms *Sipunculus nudus* on the composition of prokaryotic communities in a tidal flat as revealed by 16S rRNA gene sequences

**DOI:** 10.1002/mbo3.802

**Published:** 2019-02-07

**Authors:** Junwei Li, Ruiping Hu, Yongjian Guo, Suwen Chen, Xiaoyong Xie, Jian G. Qin, Zhenhua Ma, Changbo Zhu, Surui Pei

**Affiliations:** ^1^ Key Laboratory of South China Sea Fishery Resources Exploitation & Utilization of Ministry of Agriculture of China, Guangdong Provincial Key Laboratory of Fishery Ecology and Environment South China Sea Fisheries Research Institute, Chinese Academy of Fishery Sciences Guangzhou PR China; ^2^ Guangzhou Haiwei Aquatic Science and Technology Co., Ltd Guangzhou PR China; ^3^ College of Sciences and Engineering, Flinders University Adelaide, SA Australia; ^4^ Annoroad Gene Technology Co., Ltd. Beijing PR China

**Keywords:** bacterial community, high‐throughput 16S rRNA gene sequencing, sandy tidal flat, *Sipunculus nudus* Linnaeus

## Abstract

To understand the impacts of peanut worms *Sipunculus nudus* on the prokaryotic community composition in a tidal flat, an onsite investigation was conducted in Suixi in the Beibu Gulf (109.82E, 21.35N) in the burrow sediments, non‐burrow sediments and the sediments without peanut worm disturbance (control). The16S rRNA gene Illumina MiSeq sequencing was used to investigate the microbial communities and their response to bioturbation by *S. nudus* in a sandy tidal flat. A total of 18 bacteria phyla were detected, and Proteobacteria and Cyanobacteria constituted the majority of the prokaryotic community in the samples. The distribution of the relative abundances of genera showed that approximately 6.99%–17% of the reads in the samples were classified into 25 known genera. Sulfate‐reducing bacteria (*Desulfococcus* and *Desulfosarcina*) were the most abundant taxa, followed by Thermodesulfovibrionaceae *LCP‐6*, indicating that sulfate reduction is the main process in the sandy tidal flat. The abundances of *Desulfococcus*, *LCP‐6 *and *Cyanobacterium* in the non‐burrow sediment were greater than in the burrow sediment, suggesting that the anoxic condition is more suitable for *Desulfococcus *and *LCP‐6* when the activity of *S. nudus* is absent. The biomass of *Cyanobacterium *was decreased by the feeding bioturbation of *S. nudus*. Meanwhile, the relative abundance of the Bacteroidetes *Luteimonas* in the burrow sediments was significantly greater than in the non‐burrow sediment, and there was a strong relationship between *S. nudus* bioturbation and increased in oxygen contents and oxidation‐reduction potentials in the burrow sediment. The abundances of *Desulfococcus* and *LCP‐6 *were greater in the middle layer (20–30 cm) than in the top layer in the non‐burrow sediment. However, the middle and bottom layers (20–30, 30–40 cm) had higher abundances of these genera than did the upper layers (0–10, 10–20 cm) in the burrow sediments. The abundances of the Fusobacteria *Propionigenium *and the Spirochaetes *Spirochaeta* were greater in the middle and bottom layers (20–30 cm, 30–40 cm) than in the top layers (0–10, 10–20 cm) in the burrow sediment, but this phenomenon was not found in the non‐burrow sediment. This study demonstrates that bioturbation by *S. nudus *plays an important role in reshaping the bacterial community composition in intertidal regions.

## 1 INTRODUCTION

Bacterial communities in the sediment play important ecological and biogeochemical roles in tidal flat ecosystems for nutrient recycling and pollutant degradation (Osborn, Bruce, Strike, & Ritchie, 1997; Stevens, Brinkhoff, Rink, Vollmers, & Simon, 2007). Chemical characteristics of the marine sediment such as organic matter, salinity, oxygen, nitrogen, and phosphorus can influence the composition and distribution of bacterial communities (Osborn, Bruce, Strike, & Ritchie, 1997; Ikenaga, Guevara, Dean, Pisani, & Boyer, 2010). In fact, bioturbation by benthic organisms through the processes of burrowing, feeding, and excretion can reshape the physicochemical properties and biological characteristics of sediments (Kristensen, 2001; Laverock, Tait, Gilbert, Osborn, & Widdicombe, 2014; Taylor & Cunliffe, 2015; Volkenborn, Hedtkamp, Beusekom, & Reise, 2007) and in turn affect benthic microbial faunas. The bacterial community and activity depend on organic content, distribution, feeding habits, and excreta of macrobenthos (Kristensen & Kostka, 2005). A previous study shows that the relative abundance of the genera *Propionigenium* of Fusobacteria, *Fusibacter *of Firmicutes, *Spirochaeta* of Spirochaetes, *Desulfococcus* of Deltaproteobacteria, and Nitrospirae *LCP*‐6 increased by the bioturbation of macrobenthos in mudflat sediments (Ma et al., 2015), and the abundance of bacteria was greater in burrow sediments than in non‐burrow sediments (Papaspyrou, Gregersen, Cox, Thessalou‐Legaki, & Kristensen, 2005). The abundance of Acidobacteria, Actinobacteria, Nitrospirae, and some other bacterial phyla can be influenced by the bioturbation of *Meretrix meretrix* and *Perinereis aibuhitensis*, dissolved oxygen and total dissolved inorganic nitrogen in sandy sediments (Shen et al., 2017), and the abundance of nitrogen‐cycling functional genes can be directly influenced by the bioturbation of *Upogebia deltaura* in coastal sediments (Laverock et al., 2014). The bioturbation of coastal shrimp can increase the abundance of bacterial communities in the burrows (Laverock et al., 2010) and stimulate nitrogen fixation (Bertics et al., 2010). In addition, *Ruditapes philippinarum *has been known to regulate benthic nitrification and denitrification in the burrow walls in coastal sediments (Welsh, Nizzoli, Fano, & Viaroli, 2015). Therefore, the biomass of macrobenthos can significantly affect the bacterial communities in the tidal flat.

Sipunculans, commonly known as peanut worms, are a group of marine non‐segmented coelomic animals in a separate phylum, Sipuncula. This phylum contains approximately 150 species (Adrianov & Maiorova, 2010, 2014; Cutler, 1994), and 40 species in this phylum are found in China (Li, Zhou, & Wang, 1992). Sipunculans are distributed worldwide from the intertidal area to the abyssal zone at different depths (Adrianov & Maiorova, 2010). Sipunculans play an important role in sediment bioturbation and are also a food source for animals at higher trophic levels (Kedra & Wlodarska‐Kowalczuk, 2008; Mark & Monika, 2009). *Sipunculus nudus* is widely distributed along the Chinese southern coasts and located in various biotopes, particularly in sandy beaches along the intertidal habitats of a seashore (Li et al., 1990; Li, Zhu, Guo, Xie, Huang et al., ). The *S. nudus *adults bury themselves into sandy substrates at a depth of 20~50 cm and they can transport organic matter from the surface into the bottom of sediments and affect microbial distribution in the sediments (Li, Zhu, Guo, Xie, Huang et al., ; Mark & Monika, 2009). However, the ecological role of sipunculid worms in reshaping bacterial community composition and biogeochemiacl cycles in intertidal zones is not well understood.

In this study, sediments from different layers were collected in a sandy tidal flat in the Beibu Gulf of the South China Sea, and the particle size, composition, organic matter, oxidation‐reduction potential (ORP), and moisture contents were determined. We aim to reveal the dominant bacterial community composition, diversity, and microbial functions along the sandy tidal flat in Beibu Gulf through high‐throughput 16S rRNA gene sequencing. Meanwhile, we investigated the response of microbial communities to the bioturbation of *S. nudus* in the sandy flat, and then established the relationship between the prokaryotic community and the physiochemical indicators in the intertidal ecosystem.

## 2 MATERIALS AND METHODS

### 2.1 Study area

**Figure 1:**
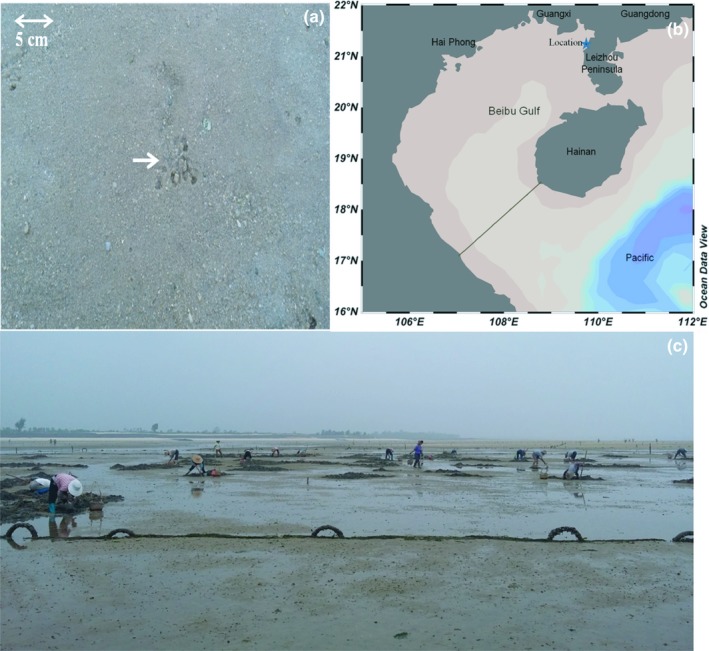
Sampling location in Beibu Gulf (a), Sipunculus nudus star‐like traces in the tidal flat (b), The aquaculture area (top) and sampling location (bottom) (c)

The study area (109°48′24.18″E, 21°21′20.70″N) was in the tidal flat in the eastern region of Beibu Gulf (Figure 1). Approximately 1,333 ha of tidal flat was used for *S. nudus* farming, and the area belonged to middle and low tidal zones. The period of low tide was less than 6 hr. The surface sediment of the tidal flats primarily consisted of quartz sand (as high as 93%), and most of the sand grain size dimensions were less than 0.85 mm (Table 1).

**Table 1:** Environmental parameters of the samples in the tidal flat

Samples	Grain size (0.1–0.18 mm) %	Grain size (0.18–0.85 mm) %	Organic content %	oxidation‐reduction potential (mv)
Sediment1	9.52 ± 1.35^b^	58.16 ± 7.32	8.50 ± 0.94	−23.00 ± 4.00^a^
Burrow1	5.22 ± 0.73^a^	66.93 ± 7.11	8.03 ± 0.78	−11.00 ± 2.00^b^
Sediment2	7.63 ± 0.52^b^	68.66 ± 6.83	7.50 ± 0.93	28.00 ± 4.00^b^
Burrow2	6.07 ± 1.03^a^	69.50 ± 8.98	7.89 ± 0.75	−67.00 ± 7.00^a^
Sediment3	5.75 ± 0.63	59.22 ± 6.45	7.90 ± 0.59	35.00 ± 6.00^b^
Burrow3	5.15 ± 0.59	68.73 ± 7.08	7.99 ± 0.93	−16.00 ± 3.00^a^
Sediment4	4.23 ± 0.41	58.24 ± 8.47	7.60 ± 0.73	2.00 ± 1.00^b^
Burrow4	5.26 ± 0.67	69.31 ± 10.29	7.20 ± 0.85	−10.00 ± 2.00^a^
Control	6.37 ± 0.69	61.35 ± 6.75	7.56 ± 0.62	12.00 ± 3.00^b^

Note

The values are presented as the mean ± *SD* (*n* = 3). The different letters indicate significant differences between the same layers in the Sediment and Burrow groups (*p < *0.05).

### 2.2 Experiment setup and sampling

Three cores were drilled in parallel in each of the burrow, sediment, and control areas in the sandy tidal flat (Figure 1). The distance from the beach to sub‐tidal zone was about 1.8 km during spring tidal, and the sampling sites were set along the coastline. The sampling zone was located in the aquaculture area of *S. nudus* (66,600 m^2^) where the same management method was applied. The sediments were derivived from three distinctive types (burrow sediment, non‐borrow sediment, and control sediment) with three replicates of each type. The burrow sediment was from the area where the worms were farmed and worm burrow activities occurred. The non‐borrow sediment was from the area where worms were distributed, but there was no burrow activity. The control zone sediment (Control) was collected from the area where no *S. nudus *was found. The samples from burrow and non‐burrow zones at the depths of 0–10, 10–20, 20–30, and 30–40 cm were labled as burrow 1, 2, 3, and 4, and sediments 1, 2, 3, and 4, respectively. The sediment cores in the control zone were not distinguished by layers (0–40 cm). All the samples were then divided into two parts and were stored into an insulated incubator with dry ice and were then immediately transported to the laboratory. One part of the samples was used for physiochemical analyses, such as particle size composition, moisture, organic matter, sandy content, and oxidation‐reduction potential (ORP). Particle size composition was obtained through a fine sieve filtration method. Organic matter content was measured using the combustion method at 550°C. ORP was measured by using an oxidation reduction potentiometer (SX 712; Sanxin Instrument Corporation, Shanghai, China) after the pore water from different layers was filtered. The other samples were used to determine the bacterial community composition according to 16S rRNA gene.

### 2.3 DNA isolation and 16S rRNA gene library preparation

DNA was extracted from sediment samples using MoBio PowerSoil^®^ DNA Isolation Kit according to the SDS method (Clegg & Griffiths, 1997). Each replicated sample (5 g) was extracted in triplicate, and the DNA samples were subsequently pooled. The concentrations of DNA were determined by using a NanoPhotometer spectrophotometer and a Qubit 2.0 Fluorometer (Life Technologies, CA). The different regions of the 16S rRNA gene were chosen and then amplified by the corresponding primers: 338F‐533R for the V3 regions, 341F‐805R for V3+V4 regions, and 967F‐1046R for the V6 regions. The V3‐V4 region of the 16S rRNA gene was amplified from the bacterial DNA by polymerase chain reaction (PCR) using the modified primers 341F (5′‐CCTACGGGNGGCWGCAG‐3′) and 805R (5′‐GACTACHVGGGTATCTAATCC‐3′) as described elsewhere (Vasileiadis et al., 2012), which was based on the design method described previously (Wang & Qian, 2009). The index sequences were added, and enrichment after the extraction was completed. The Qubit 2.0, Agilent 2100 and Bio‐Rad CFX 96 instruments were used to quantify the concentration and purity of the library samples to ensure their quality. After these steps were complete, the library was sequenced on an Illumina MiSeq platform by using the 250 paired‐end (PE) protocol.

### 2.4 Illumina MiSeq sequencing

All reads completely matching the barcodes with a maximum of a single mismatch to the primers were retained and then trimmed by removing the sequencing adaptor, barcodes and primer sequences to obtain valid raw reads. The barcoded Illumina MiSeq paired‐end sequencing (BIPES) pipeline was used to process the raw sequences and generate overlapped tags (Bartram, Lynch, Stearns, Moreno, & Neufeld, 2011; Zhou et al., 2011). The software QIIME was used to analyze the data received from Illumina PE sequencing (Caporaso, Kuczynski, Stombaugh, & Bushman, 2010). Briefly, the PE reads were separated from each sample according to their barcode sequence (Bokulich et al., 2013). All of the sequences that contained one or more ambiguous reads or mismatches in the primer sequences were removed during the overlap step, and reads with complete barcode sequences were selected for subsequent analysis. Afterward, the clean sequences were screened for chimeras using UCHIME (Edgar, Haas, Clemente, Quince, & Knight, 2011). Prior to cluster analysis, the paired read sequences were merged using the PEAR software (Zhang, Kobert, Flouri, & Stamatakis, 2014). To generate taxonomic profiles of the sediment samples included in this study, all the OTUs with a 97% similarity were clustered using UCLUST software (Edgar, 2010); and then, the representative sequence of each OTU was compared against the Greengene taxonomic database using PyNAST (Caporaso et al., 2010).

### 2.5 Statistical analysis

The microbial alpha diversity was analyzed using the number of observed species, the Chao I (Chao, 2002), Shannon estimators (Magurran, 1988), and Simpson's index. The phylogenetic diversity (PD) whole tree method (Faith, 1992) was used to analyze the relationships among observed species by the PyNAST method (Caporaso et al., 2010) and GraPhlAn software (Langille et al., 2013). Beta diversity analysis of microbial communities was performed with principal component analysis (PCA) and principal coordinate analysis (PCoA) based on a matrix of operational taxonomic units (OTUs). The PCoA was taken into account for the matrix of weighted or unweighted UniFrac distances (Lozupone & Knight, 2005). Both PCA and PCoA were performed using the QIIME software (qiime‐1.80) and displayed using R software. The adonis test was used in PCoA analysis and the diversity analysis was mainly based on QIIME v1.80. QIIME and rdp‐classifier v2.2 were adopted to compare the assembly tags aligned with Greengene 13.8, and the tags with a 97 percent similarity were classified as the same OTU. Data were analyzed using SPSS 13.0 for Windows statistical software. The effect of bioturbation on environmental indicators and microbial diversity was analyzed using one‐way ANOVA (*n* = 3). The differences between the same layer in the sediment and burrow groups were analyzed, and differences were considered significant at *p* < 0.05.

## 3 RESULTS

### 3.1 Environmental indicators

The environmental parameters from the sampling site are shown in Table 1. The salinity was 31‰, pH was 8.29 and temperature was 25°C. The grain size of 0.10–0.18 mm in the sediment decreased with the increase of sampling depth, while was not significantly different between layers within the burrow sediments. The particle size of 0.10–0.18 mm was higher in non‐burrow sendiments 1 and 2 than in the burrow sediments 1 and 2, respectively (*p < *0.05, Table 1). The sandy contents of the samples were higher than 90%, and the organic content was 7.60%–8.50% in non‐burrow sediment, and 7.20%–8.02% in the burrow sediments without any significant difference between the two groups. The ORP value was greater in burrow 1 than in sediment 1, and the values were lower in burrow sediments 2, 3, and 4 than in non‐burrow sediments 2, 3, and 4, respectively (*p < *0.05, Table 1).

### 3.2 Microbial diversity

In total, 984,268 raw reads were generated from the samples. After filtering, 901,334 (91.57% of the total reads) were used in the analysis. A total of 448,398 pairwise reads were generated from the nine sediment samples, and the number of effective tags generated from the nine sediment samples ranged from 11,483 to 17,272 with a total of 130,965. All the valid sequences were used for further analysis of bacterial composition and diversity analysis.

**Figure 2:**
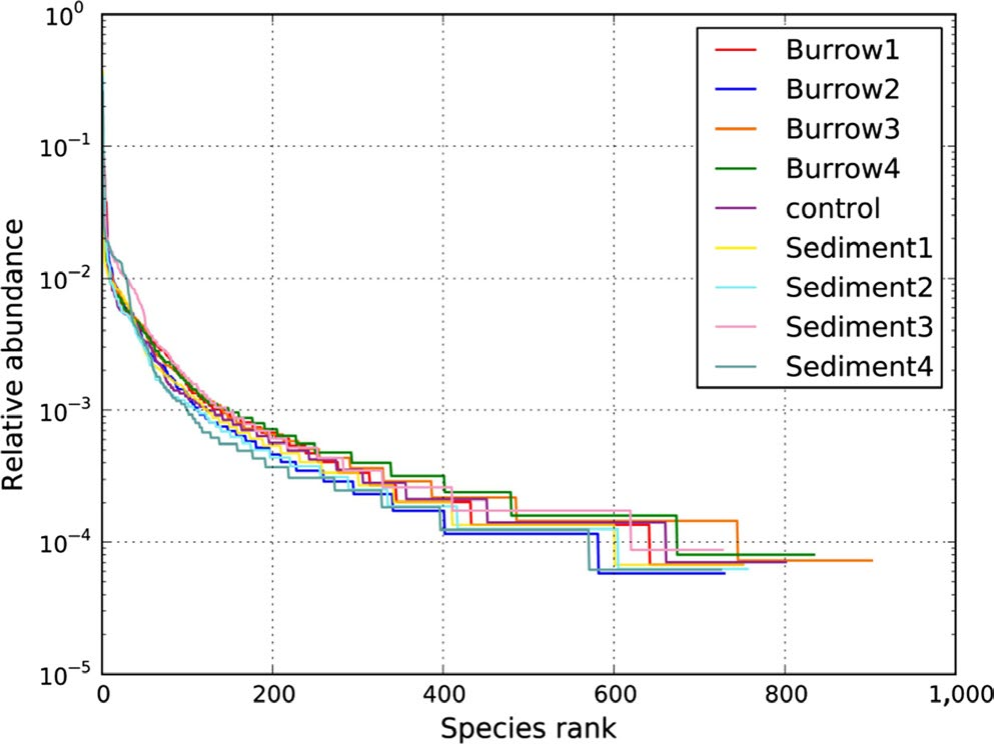
Relative abundance of ranking OTU sequences in the samples

The alpha diversity indicators the Chao1 estimator, the number of observed species, and the Shannon and Simpson's indexes are shown in Table 2. The relative abundances of OTUs are displayed in Figure 2. The index value and slowly declining trend show that the nine sediment samples had high species richness and bacterial diversity. Approximately 725 to 902 OTUs were found in the non‐burrow sediments, and the amount of data was sufficient for further analysis. The number of OTUs was higher in the sediment burrows 3 (20–30 cm) and 4 (30–40 cm) than in the non‐burrow sediment (*p < *0.05), and the burrow sediment 3 had the highest number of OTUs and bacterial species. However, non‐burrow sediment 3 (20–30 cm) had the highest Shannon index and Simpson's index (Table 2). A correlation with *R* ^2^ = 0.94 was found between Chao1 and the number of OTUs. The Shannon and Simpson's index are greater in the burrow sediment than in the non‐burrow sediment. The number of observed species and Shannon index of bacteria were greater in the middle and lower layers (20–30 cm, 20–40 cm) than in the upper layers (0–20 cm). In the present study, the average Shannon index was lower in the non‐burrow sediments (6.35) than in the burrow sediments (6.75). In addition, the microbial diversity was evenly distributed in the four layers of the *S. nudus* burrows (0–10, 10–20, 20–30, 30–40 cm). However, this index varied among the four layers of non‐burrow sediments.

**Table 2:** Averaged alpha diversity indicators: the Chao1 estimator, number of observed species, and the Shannon and Simpson's index in sediment

Samples	Chao1	Observed species	Shannon	Simpson
Sediment 1	831.41 ± 16.35	685.00 ± 16.00	5.93 ± 0.41^a^	0.87 ± 0.07
Burrow 1	807.57 ± 15.61	696.00 ± 16.00	6.91 ± 0.51^b^	0.97 ± 0.06
Sediment 2	843.52 ± 20.91^b^	676.00 ± 14.00^b^	5.77 ± 0.56	0.88 ± 0.06
Burrow 2	803.66 ± 15.36^a^	635.00 ± 13.00^a^	6.16 ± 0.43	0.92 ± 0.07
Sediment 3	764.51 ± 13.28^a^	715.00 ± 18.00^a^	7.52 ± 0.68	0.99 ± 0.09
Burrow 3	988.19 ± 20.44^b^	845.00 ± 14.00^b^	6.94 ± 0.46	0.94 ± 0.06
Sediment 4	817.16 ± 17.27^a^	646.00 ± 15.00^a^	6.16 ± 0.63	0.93 ± 0.08
Burrow 4	910.47 ± 18.32^b^	801.00 ± 12.00^b^	6.98 ± 0.70	0.95 ± 0.07
Control	876.91 ± 26.38	747.00 ± 12.00	6.22 ± 0.57	0.89 ± 0.08

Note

Sediment1, 2, 3 and 4 represent the layers of 0~10 cm, 10~20 cm, 20~30 cm and 30~40 cm in sediment without *Sipunculus nudus* burrows in the tidal flat. Burrow1, 2, 3 and 4 represent the layers of 0~10 cm, 10~20 cm, 20~30 cm and 30~40 cm in the burrow of *S. nudus*, respectively. Control represents the non‐aquaculture zone. The values are presented as the mean ± *SD* (*n* = 3). The different letters indicate significant differences between the same layers in the Sediment and Burrow groups (*p < *0.05).

### 3.3 Taxonomic composition at the phylum levels

**Figure 3:**
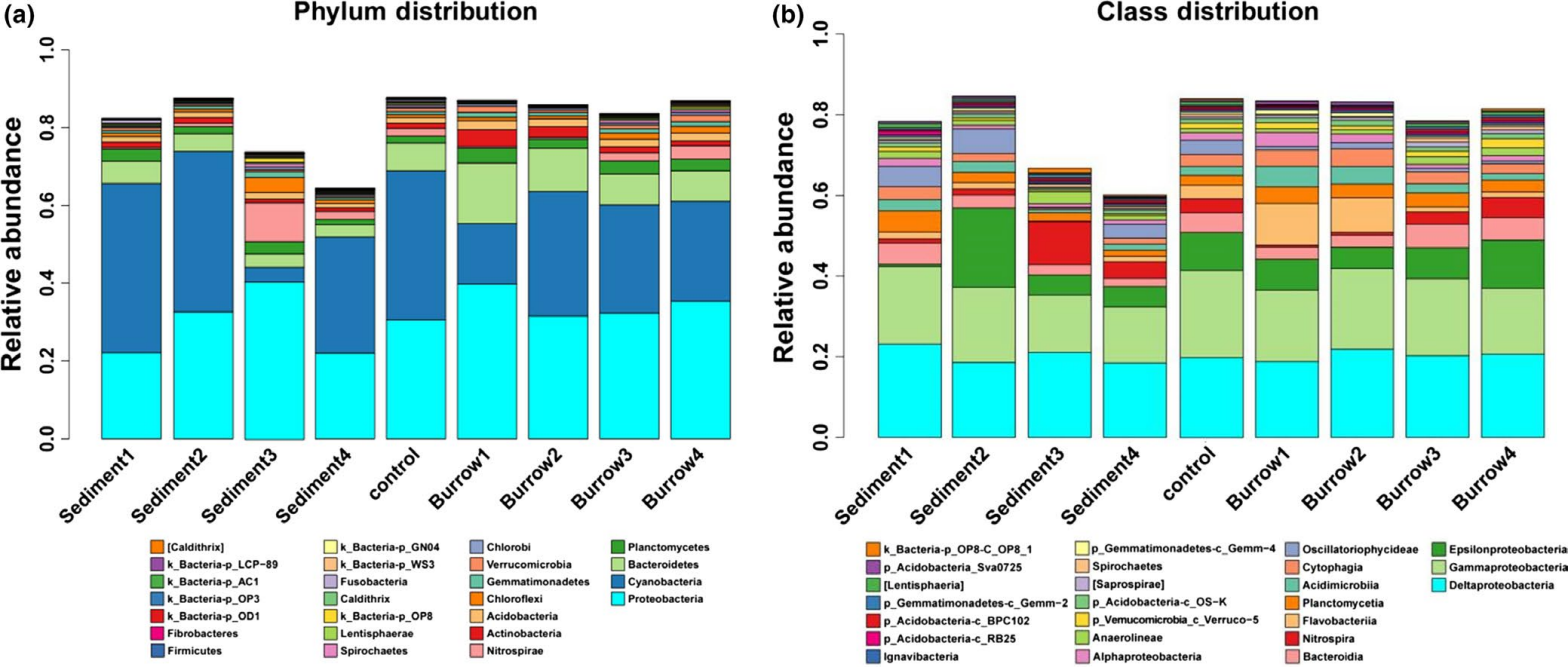
Bacterial classification at the phylum level (a) and the class level (b)

During the study period, 18 bacteria phyla were detected in the nine samples and all the reads were classified into bacterial phyla. Proteobacteria and Cyanobacteria comprised the majority of the microbial communities in the samples, and Bacteroidetes, Planctomycetes, Nitrospirae, Actinobacteria, Acidobacteria, Chloroflexi, Gemmatimonadetes, Verrucomicrobia, Chlorobi, Spirochaetes, Lentisphaerae, Calditrichaeota, Fusobacteria, Firmicutes, and Fibrobacteres were also found in the samples (Figure 3a). In the non‐burrow sediments, Cyanobacteria was the most dominant phylum in the layers of 0–10 cm (43.5%) and 10–20 cm (41.3%), and Proteobacteria was the most dominant at the depth of 20–30 cm (40.4%). The abundance of Actinobacteria was lower in the bottom layers (20–30, 30–40 cm) than in the upper layers (0–10 and 10–20 cm). However, the abundances of Nitrospirae, Chloroflexi, Gemmatimonadetes, Chlorobi, Spirochaetes, Caldithrix, and Firmicutes were greater in the layers of 20–30 and 30–40 cm than in the upper layers (0–10, 10–20 cm). In burrow sediments, Proteobacteria was dominant in the four layers (39.7%, 31.5%, 32.3%, 35.3%), and Cyanobacteria accounted for 15.5%, 32.0%, 27.8%, and 25.7% in the four layers (0–10, 10–20, 20–30, 30–40 cm), respectively. As found in the non‐burrow sediments, Nitrospirae (0.49%, 0.82%, 10.08%, 2.19%), Chloroflexi (0.89%, 0.76%, 3.89%, 0.86%) and Spirochaetes (0.19%, 0.20%, 0.86%, 0.29%) were greater in the layer of 20–30 cm than in the upper layers, and the abundance of Actinobacteria (1.38%, 1.52%, 0.89%, 0.87%) was lower in the bottom layers (20–30, 30–40 cm) than in the upper layers. The average abundances of Proteobacteria, Bacteroidetes and Actinobacteria were greater in burrow sediments than that in non‐burrow sediments (*p < *0.05), and the abundance of Cyanobacteria (43.54%) was greater in the surface of non‐burrow sediments than that in each layer of the burrow sediments (15.5%, 32.0%, 27.8%, 25.7%, *p < *0.05). In addition, the Nitrospirae in non‐burrow sediment 3 was significantly higher than the burrow sediments (*p < *0.05).

### 3.4 Taxonomic classification at the family and genus levels

**Figure 4:**
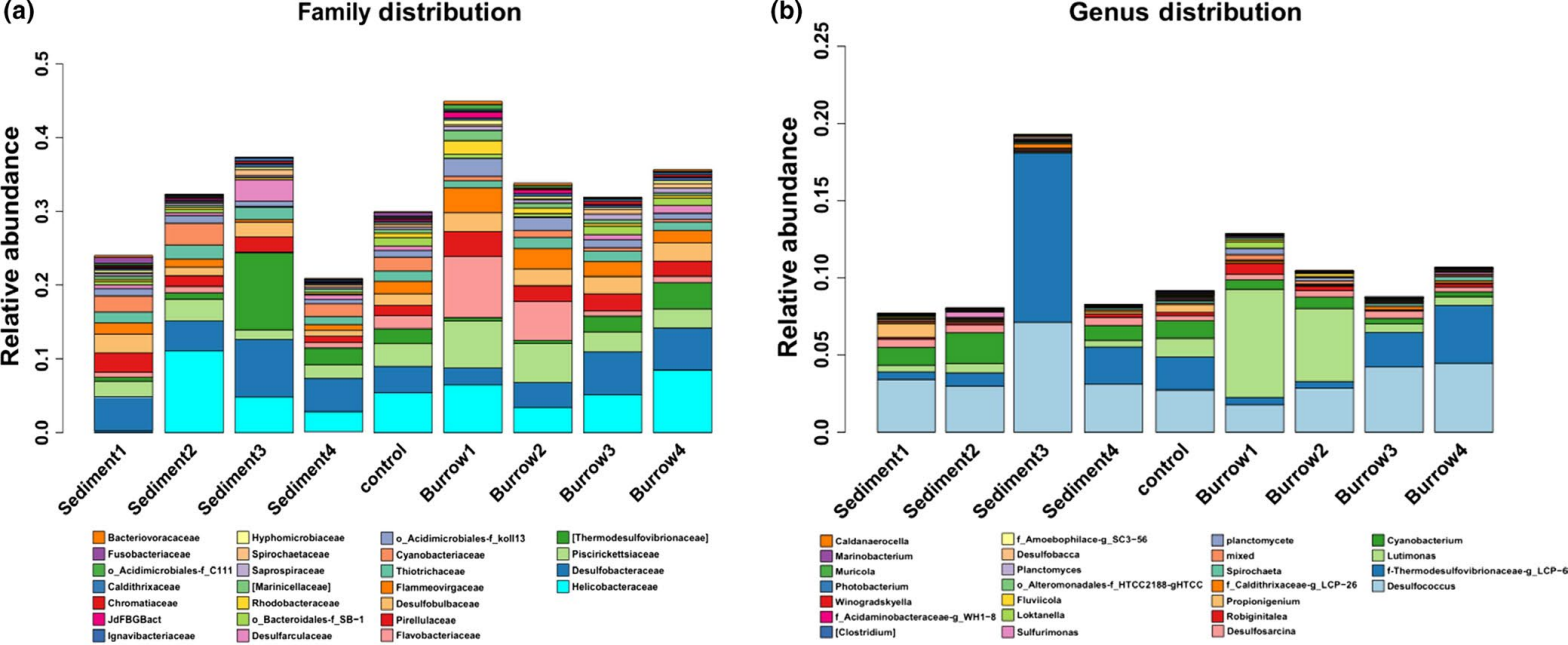
Bacterial classification at the family level (a) and genus level (b)

The distribution of relative abundances at the family level showed that approximately 20%–43% of the reads in the samples were classified into 25 known families (Figure 4a). In non‐burrow sediments, the community composition of bacteria showed a sequence of bacterial abundance of Desulfobacteraceae (5.07%) > Helicobacteraceae (4.57%) > Thermodesulfovibrionaceae (3.38%) > Piscirickettsiaceae (1.99%) > Cyanobacteriaceae (1.69%) > Pirellulaceae (1.67%) > Desulfobulbaceae (1.59%) > Thiotrichaceae (1.49%) > Desulfarculaceae (1.1%). Desulfobacteraceae was abundant in the four layers of sandy sediment (0–40 cm), and the abundance of Thermodesulfovibrionaceae and Desulfarculaceae was higher in the layer of 20–30 cm than in the other layers. In addition, the abundances of Piscirickettsiaceae, Flammeovirgaceae and Cyanobacteriaceae were lower in the bottom layers (20–30, 30–40 cm) than in the top layers (0–10, 10–20 cm). In the burrow sediments, Helicobacteraceae, Desulfobacteraceae, and Piscirickettsiaceae were dominant in all the layers, and the community composition of bacteria showed the order of abundance as Helicobacteraceae (5.68%) > Desulfobacteraceae (4.14%) > Piscirickettsiaceae (4.1%) > Flavobacteriaceae (3.66) > Flammeovirgaceae (2.39%) > Desulfobulbaceae (2.33%) > Thermodesulfovibrionaceae (1.58%) > Thiotrichaceae (1.19%). Notably, the abundances of Desulfobacteraceae, Thermodesulfovibrionaceae and Thiotrichaceae were greater in the bottom three layers than in the top layer (0–10 cm), but Piscirickettsiaceae, Flavobacteriaceae and Flammeovirgaceae were greater in the top layers (0–10, 10–20 cm) than in the low layers (20–30, 30–40 cm). Most of the abundances of the bacteria were greater in the burrow sediment than in the non‐burrow sediment.

The distribution of the relative abundances at the genus level showed that approximately 6.99%–17% of the reads in the samples were classified into 25 known genera (Figure 4b). In the non‐burrow sediments, the community composition of bacteria showed the sequence of abundance as *Desulfococcus (*3.77%) > *LCP‐6* (3.55%) > *Cyanobacterium* (0.93%) > *Desulfosarcina *(0.37%) > *Luteimonas *(0.34%) > *Propionigenium *(0.27%) > *LCP‐26 *(0.14%) > *Robiginitalea* (0.11%) > *Sulfurimonas* (0.109%). *Desulfococcus *was dominant in the sandy sediment, and its abundance was higher in the layer of 20–30 cm than in the other layers. The abundance of *LCP‐6 *was greater in the two lowest layers than in the top two layers, but the abundance of *Cyanobacterium* was higher in the top layers than in the bottom layers. In the burrow sediments, the sequence of abundance was *Desulfococcus *(3.03%) > *Luteimonas *(2.92%)> *LCP‐6* (1.56%) > *Cyanobacterium* (0.46%) > *Desulfosarcina* (0.36%) > *Robiginitalea* (0.28%) > *Propionigenium* (0.26%) > *Planctomycete *(0.15%) > *LCP‐26* (0.13%) > *Spirochaeta *(0.127%). Some other genera were also found in the samples, including *Loktanella*, *Fluviicola*, *Planctomyces*, *Helicobacter*, *Clostridium,* and *Marinobacterium*. The abundances of *Desulfococcus*, *LCP‐6*, *Propionigenium *and *Spirochaeta* were higher in the bottom layers (20–30, 30–40 cm) than in the surface layers (0–10, 10–20 cm). In addition, the abundances of *Luteimonas*, *Cyanobacterium*, *Loktanella* and *Fluviicola *were greater in the top layers (0–10, 10–20 cm) than in the bottom layers (20–30, 30–40 cm) in burrow sediments, and the species of bacteria that were dominant in the upper layers (0–10, 10–20 cm) were more abundant in the burrow sediments than in the non‐burrow sediments. Notably, the abundance of Luteimonas was greater in the burrow sediments than in the non‐burrow sediments and was significantly greater in the top two layers (0–10, 10–20 cm) than in the other samples.

### 3.5 Heatmap of the abundant bacteria in all samples.

**Figure 5:**
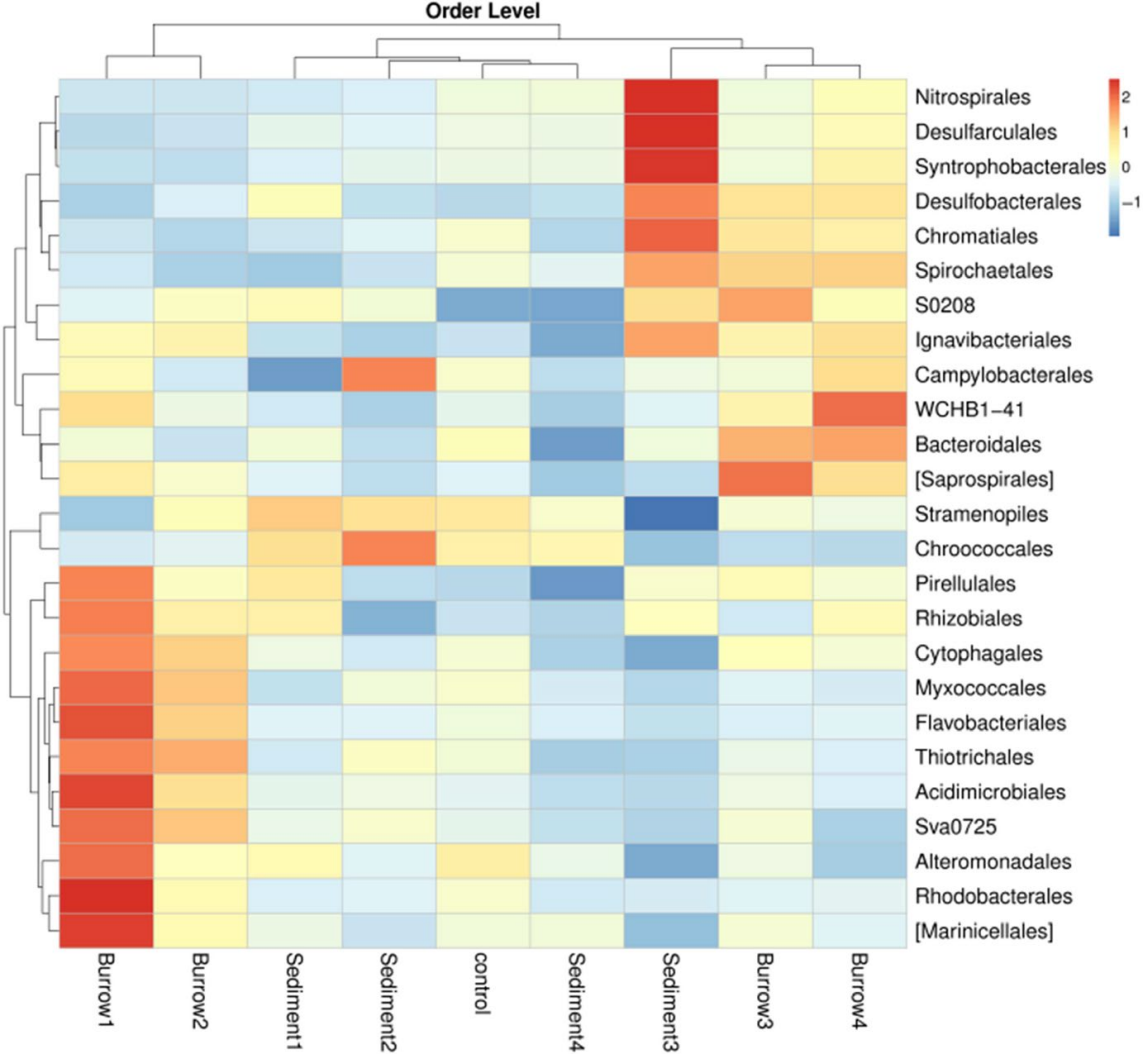
Heatmap of the 25 most abundant bacterial orders in all samples based on 16S rDNA‐based taxonomic identities of bacteria. The color scale on the right (2 to ‐1) represents the relative intensity of each bacterial order: red represents the highest abundance, and blue represents the lowest abundance

The 25 most abundant bacteria were analyzed, and the results are shown in Figure 5. Vertical clustering indicates the similarity of bacteria in the samples, i.e., the closer the distances and the shorter the branches are, the more similar the bacterial community composition and the relative abundances of the bacteria. Generally, the relative abundance of bacteria decreases with increasing sediment depths in the heatmap at the order level (Figure 5). In the burrow sediments, the bacterial communities of burrows 1 and 2 showed high similarity and were classified into the same cluster, but burrows 3 and 4 were classified into another cluster though there were significant differences between the two clusters. The layers of 0–10 and 10–20 cm were more likely to maintain oxygen‐rich conditions. Overall, in the non‐burrow sediment, the bacterial communities of sediments 1, 2, 4 and the control showed high similarity and were classified into the same cluster. However, the bacterial community of sediment 3 is different from that of the other cluster.

### 3.6 Statistical comparison of the 16S amplicons among the samples

**Figure 6:**
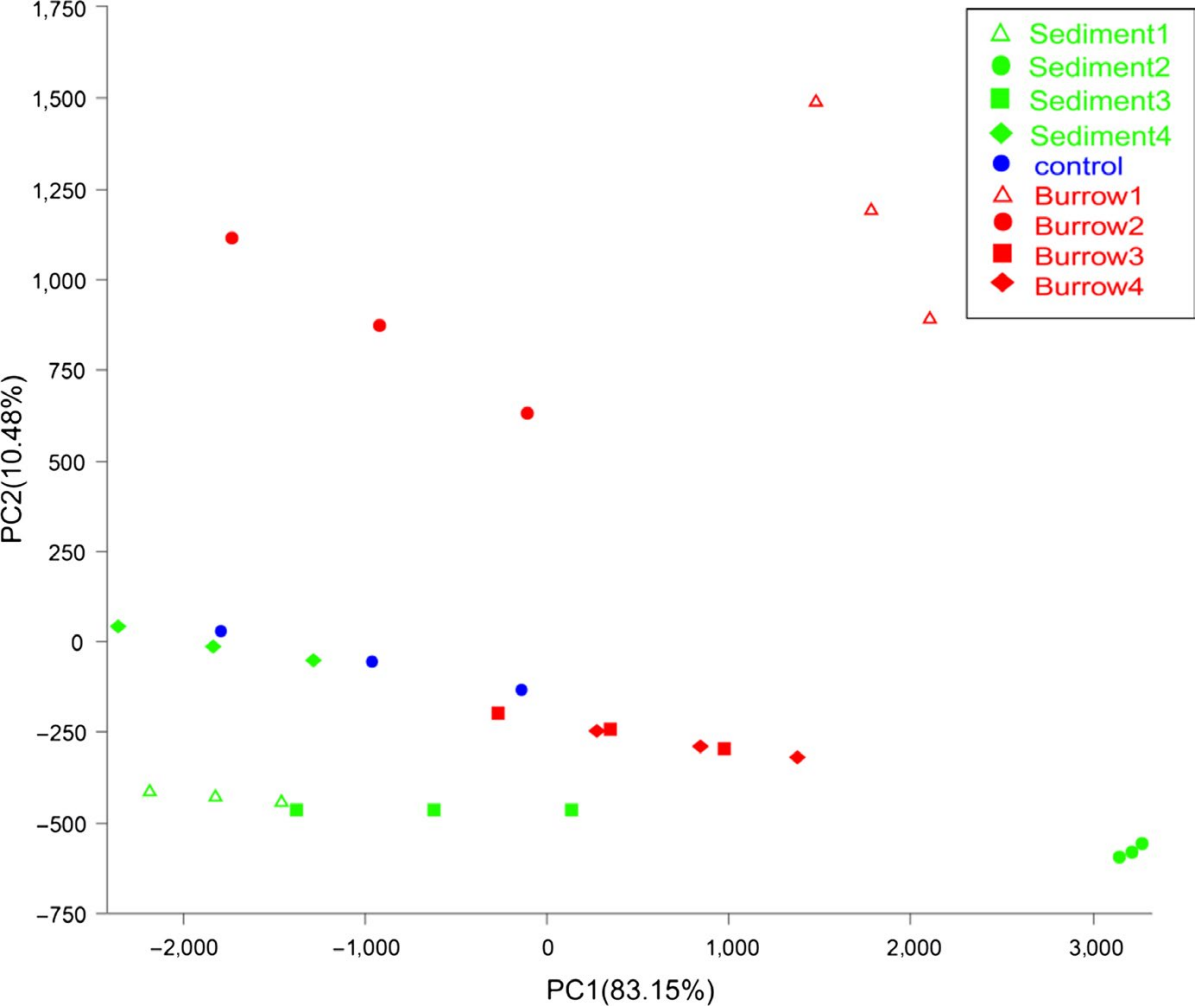
Principal coordinate analysis results with weighted UniFrac metric

In this study, PCoA1 and PCoA2 represent the factors that affect the dispersal of groups, and they explain the difference of the 83.15% and 10.48%, respectively (Figure 6). The samples were classified into three groups. The non‐burrow sediments 1 and 4, burrow sediment 2 and the control were grouped tightly. However, the non‐burrow sediments 2 and 3 were not grouped with the other samples. The burrow sediments 3 and 4 were grouped tightly (Figure 6). Generally, there were differences in prokaryotic community composition between non‐burrow sediments and burrow sediments, but only the subsurface layer (10–20 cm) in the burrow sediment was similar to that of the non‐burrow sediments 1 and 4 (0–10, 30–40 cm). For the change of prokaryotic community composition with depth, the compositions in non‐burorw sediments 2 (10–20 cm) and 3 (20–30 cm), were very different from the other layers (0–10, 30–40 cm). The bottom layers (20–30 and 30–40 cm) in the burrow sediments had the similar prokaryotic community composition, and there was some difference between the surface (0–10 cm) and subsurface (10–20 cm) in the burrow sendiments.

## 4 DISCUSSION

### 4.1 Prokaryotic community structure in sandy tidal flat in the Beibu Gulf

Tidal flats are an environment that experiences periodical change due to daily tidal cycles, resulting in gradients along physical and chemical parameters such as moisture, temperature, nutrients, and salinity (Menge & Branch, 2001). The ever‐changing environment might result in a high diversity of intertidal microbes (Ma et al., 2015). The Shannon index was slightly lower in the tidal flat of *S. nudus* than in the other intertidal sediments (Wang, Liu, Zheng, Zhu, & Wang, 2013; Zheng, Wang, & Liu, 2014). However, the Shannon index (6.94) in burrow 3 was close enough to the value in the burrow of *Meretrix meretrix* and *Perinereis aibuhitensis* in the intertidal flat (Shen et al., 2017). In the present study, the microbial diversity was relatively evenly distributed in burrows; however, the index varied greatly in the four layers of sediment samples. Bioturbation by macrobenthos can improve the homogeneity of organic matter in burrows (Fanjul, Escapa, Montemayor, Addino, & Alvarez, 2015; Nogaro, Menmillod, & Montuelle, 2007; Palmer, 2010), and it is easy to form a relatively balanced bacterial community in burrows. The Shannon index was 7.52 in non‐burrow sediment 3 (20–30 cm), which is greater than in the other layers and burrow walls. The *S. nudus* accumulates organic matter in the layer of 20–30 cm to satisfy its biological requirements (Li et al., 2018), and the high organic content and low oxygen content are probably responsible for the high bacterial diversity in the layer of 20–30 cm in the non‐burrow sediments, which can be used to explain the differences in prokaryotic community composition between sediments 3 and the other sediment layers (Figures 5 and 6).

Most of the bacterial phyla are widely distributed throughout various environments such as sea water, sediment, and soil in the coastal regions (Hery et al., 2014). In the present study, Proteobacteria was also the most dominant phylum (Figure 3a), and a similar situation was found in other studies (Andrei et al., 2017; Ma et al., 2015; Shen et al., 2017; Zhang, Hu, Ren, & Zhang, 2018; Zhu, Wang, Zhang, Zhu, & Zou, 2016). However, the total number of bacterial phyla was similar to that in sandy tidal flat (18 phyla), wetland (20 phyla) and early biofilms (11 phyla) (Peng, Li, Lu, Xiao, & Yang, 2018; Shen et al., 2017; Zhu et al., 2016), but less than that in mudflats (53), hypersaline sapropels (59 phyla) and mangrove mudflats (57 phyla) (Ma et al., 2015; Andrei et al., 2017; Zhang, Hu, Ren, & Zhang, 2018), most likely because of the differences in the physicochemical properties and disturbances in the environments. The main reason for these relationships might also be that the organic matter content was lower, and the oxygen content was higher in the sandy tidal flat than in the other mudflat habitats.

### 4.2 Effects of benthos bioturbation on the microbial communities in tidal flat sediment

Macrobenthos bioturbation processes such as burrowing, feeding, and excretion reshape the physicochemical properties and biological characteristics of the sediment (Kristensen, 2001; Volkenborn et al., 2007) and affect the bacterial community composition. The bacterial community composition and metabolism mainly depend on the organic content, particle size distribution, feeding habits, and excreted substances (Kristensen & Kostka, 2005). The *S. nudus *has a clear ecological function of transporting organic matter from the surface layer into the bottom sediments (Li, Zhu, Guo, Xie, Huang et al., ), and plays an important role in the transfer of organic matter and bacterial communities in the sediment. Previous studies found that the relative abundances of the genera *Propionigenium* of Fusobacteria, *Fusibacter *of Firmicutes, *Spirochaeta* of Spirochaetes, *Desulfococcus* of Deltaproteobacteria, and Nitrospirae *LCP*‐6 are increased by the bioturbation of the macrobenthos in mudflat sediments (Ma et al., 2015) and that the abundance of bacteria was greater in burrows than in oxygen‐poor sediments (Papaspyrou et al., 2005). Proteobacterieae includes alpha‐, beta‐, delta‐, and gamma‐proteobacteria, and many kinds of microbes play an important role in sulfate‐reducing or nitrogen‐fixing (Ma et al., 2015). *Desulfococcus* members are known to play an important role in sulfate reduction, and they can convert SO_4_^2‐ ^to H_2_S under anaerobic conditions (Ravenschlag, Sahm, Knoblauch, Jørgensen, & Amann, 2000). Nitrospirae *LCP‐6* belongs to *Thermodesulfovibrio*. This group of bacteria is also associated with sulfate reduction and organic compounds’ degradation (Daims, 2014). In the present study, the Deltaproteobacteria *Desulfococcus *was dominant in the sandy tidal flat, and the abundances of *Desulfococcus* and Nitrospirae *LCP‐6 *were greater in the non‐burrow sediment than that in the burrow sediments (Figure 4b). These may indicate that the microenvironment (oxygen‐poor condition) of sediment samples is more suitable for the sulfate‐reducing bacteria than the existing burrow of *S. nudus*. The obvious disturbation by *Pestarella tyrrhena* such as wide burrow, multiple branches, constantly digging‐filling and selective feeding can lead to the similar bacterial community between burrows and ambient sediments (Papaspyrou, Gregersen, Cox, Thessalou, & Kristensen, 2005). In the narrow and straight burrows, smooth activity and feeding inside may be the main reasons for the different bacterial communities between burrows and ambient sediments. Cyanobacteria, mainly in surface sediment, can carry out photosynthesis and nitrogen fixation in the presence of light and oxygen (Herrero, Muro‐Pastor, & Flores, 2001). In the present study, the Cyanophyta *Cyanobacterium *was also mainly distributed in the surface, and its abundance was greater in upper sediment than that in all the burrow samples (*p < *0.05, Figure 4b), indicating that the feeding of *S. nudus* can reduce the biomass of Cyanobacteria in the aquaculture tidal flat (Li et al., 2018). Bacteroidetes are a group of saprophytic bacteria and well‐known degraders of organic matter (Thomas, Hehemann, Rebuffet, & Michel, 2011). The Bacteroidetes *Luteimonas *is known to be a strictly aerobic genus (Yang, Choo, & Cho, 2007), and we found that Bacteroidetes *Luteimonas* was significantly more abundant in burrow sediments than in the sediment without burrow and the control. There is a relation between the bioturbation by *S. nudus *and the enhancement of oxygen content in burrows, and the ORP values also support this view.

The abundances of *Desulfococcus* and *LCP‐6* were greater in the bottom layers (20–30, 30–40 cm) of the non‐burrow sediments than in the top layers, and a similar situation was found in the burrow sediments. Meanwhile, the abundances of the Fusobacteria *Propionigenium *and the Spirochaetes *Spirochaeta* were greater in the bottom layers (20–30 cm, 30–40 cm) than in the surface layers (0–10, 10–20 cm) in burrows. *Propionigenium *can ferment succinate and organics to form propionate under oxygen‐deficient conditions, and *Spirochaeta *is the genus of free‐living saccharolytic spirochetes (Martin, Stanley, Eugene, Karl‐Heinz, & Erko, 2006). In addition, all these behaviors are related to the anoxic conditions and organic matter in the bottom layers of the sandy tidal flat. A previous study showed that the diversity of bacteria decreased with increasing depth in mangroves and that oxygen content is the major factor that influences the diversity of bacteria in sediment (Lyimo, Pol, & Camp, 2002; Zhang, Peng, & Zhang, 2007). In the present study, the underground water permeability to the burrow may be the main reason for the descent of anaerobic bacteria in upper layers. *S. nudus* can accumulate organic matter in the layer of 20–30 cm (Li et al., 2018), and this behavior may create suitable growth conditions for specific anaerobic bacteria in the lower layers. Previous studies indicate that the oscillatory character of pore‐water chemistry in the presence of hydraulically active organisms has significant effects on microbial diversity and biogeochemical processes in marine sediments (Volkenborn, Polerecky, Wethey, DeWitt, & Woodin, 2012; Volkenborn, Polerecky, Wethey, & Woodin, 2010). The *S. nudus* was a typically active organism with repeated body motions and constant extension and flexiation of body shapes during the feeding process (Li, Zhu, Guo, Xie, Huang et al., ). This characteristic would contribute to the fast process of biogeochemical cycles in sandy tidal flats.

### 4.3 Relationship between the main prokaryotic community and the physicochemical characteristics

Sulfate‐reducing bacteria (SRB, *Desulfococcus* and *Desulfosarcina*) were the most abundant microbial organisms in the present study. The Thermodesulfovibrionaceae *LCP‐6* in the Nitrospirae phylum also had a high relative abundance in the genus’ distribution, indicating that sulfate reduction was the main cycling process in the sandy tidal flat. The Desulfobacteraceae is a family of Proteobacteria, and most species of Desulfobacteraceae reduce sulfates to sulfides to obtain energy and are strictly anaerobic (Daims, 2014). SRB can be affected by sulfate content, organic content, temperature, oxygen content and the bacterial community in small niches (Wang, Liang, Yuan, Zhang, & Zeng, 2008). *Desulfococcus *can dominate environments that have a low content of organic matter and sulfate without restriction (Icgen & Harrison, 2006; Liang et al., 2003; Zhang & Zhang, 2016). The microbial activity and sulfate reduction rate can be enhanced by organic excretion from *Mya arenaria* in the burrow (Hansen, King, & Kristensen, 1996). In the present study, the abundances of *Desulfococcus*, *Propionigenium *and *Spirochaeta* were greater in the bottom layers (20–30, 30–40 cm) than in the surface layers (0–10, 10–20 cm) in burrows, which showed that the bioturbation of *S. nudus *can create better conditions for the growth of bacteria.

The SRB were mainly found in the layer of 0–20 cm in Erhai, and they were mainly found in the layer of 0–7 cm in Hongfenghu. The main reason for their abundant distribution in these locations was that there was more sulfate matter accumulated in the sediment in Erhai but no large amounts of organic matter and sulfate accumulation in the sediment in Hongfenghu (Liang et al., 2003; Zhang & Zhang, 2016). In the present study, SRB were mainly found in the layers of 20–30 and 20–40 cm in the sediment and burrows, respectively. The anaerobic condition and high content of sulfate appear to be the primary factors. Therefore, the bioturbation of *S. nudus* could affect the distribution of SRB in the sandy tidal flat. The *S. nudus *showed the phenomenon of moving away from the old burrow (Li, Zhu, Guo, Xie, Huang et al., ), and the sulfide accumulation could be a reason for their continuous migration, causing growth decline of *S. nudus* in the Beibu Gulf.

In conclusion, the bacterial community composition can be affected from the surface to the bottom layer by the bioturbation of *S. nudus*. Proteobacteria and Cyanobacteria constitute the majority of the prokaryotic community in the sandy flat in Beibu Gulf. Sulfate‐reducing bacteria were the main group in the sandy tidal flat, while the Desulfococcus and Nitrospirae Thermodesulfovibrionaceae *LCP‐6* varied significantly at the depth of 20–30 cm between burrows and sediments. The anoxic condition and rich organic matter in the deep layer might be responsible for the variation of SRB community, especially for *LCP‐6*. The accumulation of sulfide can lead to slow growth of *S. nudus* and low biodiversity in a short time. The variation of sulfate metabolism in the sediment and burrows need further study in the future.

## CONFLICT OF INTEREST

The authors declare no conflict of interest.

## AUTHORS CONTRIBUTION

L.J.W., H.R.P., G.Y.J., C.S.W., X.X.Y, Q.J.G., M.Z.H., and Z.C.B. contributed to the experiment design and sampling. L.J.W. collected the data and wrote the manuscript. M.Z.H., and Z.C.B. revised the final version of the article, P.S.R. processed the data.

## ETHICS STATEMENT

None required.

## DATA ACCESSIBILITY

The OTU data is available in the Figshare repository at https://figshare.com/s/2d74a80aeb46ca8874db.
